# Trazodone Addition to Paroxetine and Mirtazapine in a Patient with Treatment-Resistant Depression: The Pros and Cons of Combining Three Antidepressants

**DOI:** 10.1155/2016/5362485

**Published:** 2016-10-11

**Authors:** Rui Lopes, José Carlos Alves, Raquel Garcia Rego

**Affiliations:** ^1^Department of Psychiatry, Hospital do Divino Espírito Santo de Ponta Delgada, Avenida D. Manuel I, Ponta Delgada, 9500-370 Azores, Portugal; ^2^Faculty of Medicine, University of Porto, Alameda Prof. Hernâni Monteiro, 4200-319 Porto, Portugal; ^3^Ciclo Básico de Medicina, Department of Biology, University of the Azores, Rua da Mãe de Deus, 9501-801 Ponta Delgada, Portugal

## Abstract

Dual antidepressant combination for treatment-resistant depression is a strategy well supported by literature and accepted in clinical practice. Rather, the usefulness of the combination of more than two antidepressants is controversial. This may be related to the possibility of higher side-effect burden and to doubts about its pharmacological effectiveness and therapeutic advantage compared to other standard treatment options. We report a relapse of moderate-to-severe depressive symptoms with insomnia that successfully remitted after the addition of trazodone to a dual combination of paroxetine and mirtazapine (in standard effective doses) in a patient with treatment-resistant depression. We also review the literature and discuss the utility of triple antidepressant combination in treatment-resistant depression. This clinical case highlights the utility of combining trazodone as a third antidepressant for the relapse of depressive symptoms after the failure of a dual antidepressant combination. Trazodone may be advantageous in patients presenting recurrence of moderate-to-severe depressive symptoms that include sleep problems and/or insomnia and may be particularly useful when benzodiazepines are not recommended. Although its use may be controversial and associated with higher risk of side-effects, more investigation is needed to determine the efficacy and safety for triple antidepressant combinations as reliable strategies for treatment-resistant depression in clinical practice.

## 1. Introduction

Major depressive disorder is associated with a high clinical, morbidity, and disability burden [1]. The number of previous episodes and subclinical residual symptoms have been identified as main predictors of recurrence [2]. Recurrent episodes in turn have been hypothetically implicated in neurodegeneration and also with cognitive dysfunction [3]. An appropriate treatment for major depressive disorder and reduction of its burden are therefore important actual key therapeutic issues. However, treatment-resistant depression or treatment-refractory depression may assume in clinical practice a true challenge and may also have a broad definition: it can usually be presented as a failure to respond to one antidepressant or two trials with antidepressants from different pharmacological classes in adequate courses (in maximal dose for at least 6 weeks of duration), to two antidepressants in combination, or to electroconvulsive therapy (ECT) or can also be presented when there is intolerance to treatment or there is a relapse after initial response to treatment [4–6].

Current clinical and consensus guidelines recommend, in a stepwise manner, switching of the initial antidepressant after the maximal dose has failed, augmentation (adding thyroid hormone, low doses of atypical antipsychotics, or mood stabilizers), and the combination of antidepressants (antidepressant polypharmacy) [7–12]. Regarding this latter strategy, although combining two antidepressants with complementary pharmacological actions is well accepted, the triple antidepressant combination is, on the contrary, less described and more controversial [13–16]. Here, we present a relapse of moderate-to-severe depressive symptoms in a patient with treatment-resistant depression that was efficaciously treated after adding trazodone to a dual antidepressant combination consisting of paroxetine and mirtazapine (in standard doses); we also discuss the pros and cons of combining three antidepressants as a strategy in the management of treatment-resistant depression.

## 2. Case Description

A 42-year-old female patient was sent to our outpatient department by her family physician due to recurrent major depressive disorder symptoms that were not responding to treatment with paroxetine 20 mg/day for 2 months. Two years before, she suffered a major depressive disorder episode, moderate-to-severe, comorbid with panic disorder that was successfully treated with paroxetine 20 mg/day during a period of 9 months. Patient achieved full remission after 2 months and completed further 6 months of treatment. After gradually tapering paroxetine during one month, she remained euthymic in the following 12 months. More recently, the patient presented a recurrence of depressive symptoms with 3 months of evolution characterized by sadness, anxiety, anhedonia, apathy, insomnia, difficulties in carrying out work activities due to low attention and concentration, decreased sexual libido and appetite, fatigue and asthenia, and feelings of hopeless and helplessness. Patient has resumed treatment with paroxetine 20 mg/day in the previous 8 weeks by her family physician. Due to the absence of treatment response, the patient was oriented to our outpatient department.

In the first appointment, she came with her husband and said that she has been married for 20 years and has two children who were 17 and 11 years old. She has been working as an employee of a clothing factory for the last 10 years. Her husband expressed concern and worries about her depressive state and said that at home she was “always complaining about everything.” They did not associate her clinical condition with the existence of recent remarkable life events such as personal, familial, or labour problems. Nevertheless, the patient was missing days of work at her employment.

We have performed a thorough clinical evaluation. On mental state examination, the patient was oriented in time, place, and person. She presented depressed mood without suicidal ideation. Psychomotor retardation was present. No hallucinatory activity and formal or content thought disorder was detected. Insight for her morbid condition was preserved. Physical and neurological examinations were unremarkable. Routine blood assessments including full blood count (FBC), plasma glucose, and urea and electrolytes (U&E), liver function tests (LFTs), thyroid function tests (TFTs), basic urine investigation, illicit drug screening, electroencephalogram, and a cerebral computed tomography were also performed and the results were within normal limits. There were no remarkable medical or family antecedents and no personal history of substance misuse. Also, there was no previous or current personal history of manic or hypomanic symptoms nor psychiatric disease in family history.

A major depressive disorder, recurrent episode, moderate-to-severe, was diagnosed [17]. Patient was instructed to raise paroxetine to 40 mg/day (20 mg bd) and to combine mirtazapine 15 mg at bedtime. She attended regular monthly or bimonthly scheduled meetings for monitoring and to also to perform a brief cognitive-behaviour psychotherapy. After a month, although there was a slight improvement on her clinical status, the patient maintained a moderate depressive mood with intermediate and final insomnia. Consequently, mirtazapine was raised to 30 mg and after 4–6 weeks of this treatment optimization her clinical remission was verified and patient returned to work.

However, after three months of paroxetine 40 mg/day and mirtazapine 30 mg/day dual combination (after 6 weeks of remission of depressive symptomatology), a moderate-to-severe relapse of depressive symptoms, accompanied by insomnia, was verified again. Relapse is usually, by definition, the return of depressive symptoms before full remission or within the first months after remission was achieved. According to DSM-5, an interval of at least two consecutive months of full remission between a previous and a new episode must be present to be considered a recurrence [17]. In this particular case, there was a return of depressive symptoms before full remission achieved two months of length and, therefore, a relapse of the previous depressive episode must be considered.

Patient and her family were informed of the possible alternatives for managing the relapse of symptomatology including the further rising of paroxetine or mirtazapine dosage; augmentation strategies (including thyroid hormones and mood stabilizers or antipsychotics in lower doses); the addition of a third antidepressant that has sedative properties and relatively well tolerable side-effect profile, such as trazodone; or alternatively ECT. Due to the possible requirement of a concomitant hypnotic benzodiazepine or sleep-inducer to first alternatives, the patient chose with her husband the addition of trazodone for the management of moderate-to-severe relapse of depression symptomatology accompanied by insomnia. This option was also preferred in detriment of ECT. Accordingly, the patient was instructed to increase slowly the trazodone dosage (50 mg every three days at bed time) till reaching 150 mg/day. She was also told to be aware of the possible warning side-effects and to stop trazodone if serious collateral symptoms were noticed. Instead, trazodone addition was well tolerated and a remarkable improvement in sleep symptoms was verified after reaching the dosage of 150 mg/day. Remission of the remaining depressive symptoms was also achieved after 6 weeks and the patient recovered her self-esteem. Regular monitoring including the assessment of weight and blood pressure and electrocardiogram as well as repeated basic blood (plasma glucose, FBC, U&E, and LFTs) and urine evaluations was performed prior to trazodone initiation and following trazodone's uptitration. Values were within normal limits and no side-effects were also detectable or reported. After 6 months, trazodone was gradually reduced (50 mg per month) and interrupted. The patient remained asymptomatic and euthymic performing paroxetine and mirtazapine in maintenance dosages for another 12 months and did not require any further medication adjustments.

## 3. Discussion

This case illustrates a relapse of depressive symptomatology accompanied by insomnia in a patient with treatment-resistant depression already performing a dual combination of paroxetine and mirtazapine (in standard doses) that was successfully treated by adding trazodone (as a third antidepressant). In parallel with optimization or augmentation, antidepressant combination represents one of the strategies recommended in treatment-resistant depression [18]. However, there remains important gaps concerning their cost-effectiveness, longer-term benefits and also to the clinical strategies to be used when these options fail [8, 19]. Dual combination of some antidepressants has broad support in literature: selective serotonin reuptake inhibitors (SSRIs) or venlafaxine (serotonin/noradrenaline reuptake inhibitor, SNRI) + mirtazapine (noradrenergic and specific serotoninergic antidepressant, NaSSA) or mianserin (tetracyclic antidepressant, TeCA); and SSRIs plus bupropion (norepinephrine and dopamine reuptake inhibitor, NDRI) [7, 9–11, 20, 21]. Other combinations, such as monoamine oxidase inhibitors (MAOI) + tricyclic antidepressants (TCA) or SSRIs + TCA, have less support but were also formerly widely used and accepted in clinical practice [19, 22]. More controversial, the combination of more than two antidepressants is less well studied and reported [13–15, 22]. This may be associated with the possibility of a greater side-effect burden (including serotoninergic syndrome and syndrome of inappropriate antidiuretic hormone secretion, SIADH) and with higher clinically significant medication interactions secondary to polypharmacy [14]. Further, there are also doubts concerning its pharmacological advantage in comparison to other standard strategies. Notwithstanding, some cases of treatment-resistant or recurrent depression that were successfully treated with a triple antidepressant combination have been recently reported and may draw attention to the possibility of constituting an available and feasible therapeutic option (Table 1) [15, 22].

Interestingly, combining antidepressants may enhance global antidepressant efficacy through complementary and additional pharmacology mechanisms. This may constitute a valid theoretical argument that also provides support for the usefulness of this strategy in clinical practice [23]. Individually, paroxetine's antidepressant properties are dependent mainly on serotonin transporter (SERT) inhibition but also on minimal norepinephrine transporter (NET) inhibition; it also has minimal anticholinergic effects due to muscarinic type 1 receptors (M1) antagonism [24]. Mirtazapine antidepressant role depends on serotonin types 2A, 2C, and 3 receptors (5-HT2A/2C/3) antagonism and alpha2 adrenergic receptors antagonism; it also exerts histaminergic type 1 receptors (H1) antagonism (sedation and weight gain), alpha1 adrenergic receptors antagonism (orthostatic hypotension), and M1 antagonism (anticholinergic effects) [24]. Trazodone (serotonin antagonist/reuptake inhibitor, SARI) exerts its antidepressant effects through SERT inhibition, 5-HT2A/2C antagonism, and alpha2 adrenergic receptors antagonism; it also presents alpha1 adrenergic receptors antagonism (orthostatic hypotension), H1 antagonism (sedation and weight gain), and minimal anticholinergic effects [25]. Moreover, trazodone's pharmacologic effects may also depend on its active metabolite, meta-chlorophenylpiperazine (mCPP), which has affinity (mainly as an agonist) at several 5-HT receptor subtypes (including 5HT2C, 5HT3, 5HT2A, 5HT1B, 5HT1A, and 5HT1D) [26, 27]. Interestingly, mCCP agonist actions on 5-HT2A and 5-HT2C receptors might oppose trazodone antagonist actions in the same receptors and hypothetically could compromise its antidepressant effect. However, it seems that* in vivo* mCCP plasma and brain levels are less than 10% of trazodone and therefore are probably blocked by the parent compound [26–28].

Notably, adding mirtazapine to paroxetine augments paroxetine's SERT inhibition (and minimal NET inhibition) through mirtazapine's 5HT2A/5HT2C/5HT3/alpha2 antagonism. The third addition of trazodone may enhance SERT inhibition and 5HT2A/5HT2C and alpha2 antagonism contributing to maximizing further this dual combination. Also, it may represent an advantageous strategy in detriment of raising the dosage of the first or second antidepressants in maximal or supramaximal dosages. Therefore, the triple combination of paroxetine + mirtazapine + trazodone can have globally the following individual complimentary antidepressant effects: SERT inhibition (both paroxetine and trazodone); NET inhibition (paroxetine); 5HT3 antagonism (mirtazapine); and 5HT2A/5HT2C and alpha2 antagonism (both mirtazapine and trazodone). Although trazodone may be associated with particular rare side-effects such as priapism [16] or nocturnal enuresis [29], it is generally well tolerated, reduces symptoms of depression, and is not associated with weight gain or sexual dysfunction [25]. Importantly, its combination with other antidepressants, particularly SSRIs and SNRIs, has been used to minimize side-effects and resolve some tolerability problems such as anxiety or sexual dysfunction and also for targeting residual symptoms not well treated by SERT inhibition alone [23, 25]. Trazodone's complementary sedative effects through H1 antagonism are also used for improving sleep quality and insomnia which promotes a minor use of benzodiazepines [25]. This feature is useful in particular clinical situations where the use of benzodiazepines and sleep-inducers may be discouraged (such as asthma, chronic obstructive pulmonary disease, and obstructive sleep apnoea) [25].

In this particular clinical case trazodone choice was therefore influenced due to an immediate effect on sleep and consequently addressing patient's insomnia symptoms and also in the management of other depressive symptomatology. In fact, trazodone ability to improve sleep has been considered one important augment mechanism to improve the efficacy of other antidepressants [24]. The alternatives of increasing paroxetine or mirtazapine dosage could also resolve depressive symptoms but require the prescription of an additional hypnotic benzodiazepine or sleep-inducer (at least during the initial phase). Moreover, there is evidence demonstrating that the mirtazapine or trazodone sedative effects may not increase with higher dosages [30, 31], but possibly one way to potentiate their sedative effect may be combining them. Further, mirtazapine could also be discontinued when trazodone was started, as both may have identical antidepressants effects (5HT2A/5HT2C/alpha2 antagonism) [24, 25]. Notwithstanding, mirtazapine can also have an additional antidepressant effect through 5HT3 antagonism [24], therefore contributing through this way to maximizing the triple antidepressant combination of paroxetine, mirtazapine, and trazodone.

Trazodone was increased gradually in accordance with patient tolerance accompanied with appropriate clinical monitoring and particular attention to side-effects burden. Following 6 months of successful treatment and clinical remission, trazodone was reduced and stopped gradually, as recommended by NICE guidelines which consider that the augmentation agent should be stopped at first instance [9].

Despite the presumptive theoretical advantage of combining three different antidepressants with complementary pharmacological profiles, its usage in clinical practice may rather be influenced by several factors including the clinical presentation itself, the personal clinician prescription practices, and, last but not the least, the choice of a clearly informant patient on the possible available treatment alternatives. Interestingly, psychiatrists' attitude about antidepressants prescription practices in clinical settings is a matter not entirely well studied [32]. Nevertheless, a convenient knowledge of general pharmacology should be present when decision on polypharmacy is made. Accordingly, higher risk of clinically significant medication interactions associated with antidepressant polypharmacy may result from inhibition and induction of cytochrome P450 (CYP) hepatic enzymes, which may lead to antidepressant toxicity or to a subtherapeutic effect, respectively. Particularly, paroxetine's inhibition of CYP1A2 and CYP3A enzymes may result in higher plasma levels of mirtazapine; further, paroxetine also inhibits CYP2D6 that may lead to higher trazodone plasma levels. Together, these additional effects may undergo reciprocal increased antidepressant levels and subsequent toxicity such as higher risk of serotonin syndrome, although this may be rare in clinical practice [10]. Nevertheless, it has also been documented that serotonin syndrome risk might increase almost exponentially with the use of antidepressants acting with different mechanisms on the same pathway and therefore this risk should be not negligible [33].

The patient, however, did not present any signs or symptoms of serotonin syndrome and also pharmacologic interactions were presumably not verified on this clinical case due to the absence of side-effects and reported toxicity. In general, although these interactions can be pharmacologically well-predicted in clinical practice, it has been recently documented that important genetic polymorphisms associated with cytochromes enzymes may lead to variable pharmacokinetic profiles (ultrarapid or poor metabolisers) [10]. Moreover, intraindividual variability on antidepressant treatment outcomes may also be dependent on different pharmacodynamic responses (such as high functioning serotonin transporter genotypes) [34]. A recent meta-analysis has demonstrated that combining antidepressants was statistically significantly superior compared to monotherapy [35]. However, the percentage of drop-outs related to adverse events with antidepressant combination was also higher [35], which therefore may raise concerns about treatment compliance. Due to higher probability of pharmacokinetic and pharmacodynamic interactions when antidepressant polypharmacy is prescribed, more careful monitoring should be therefore a practical concern to prevent unexpected or rare side-effects. This is especially important in certain patients groups such as those with renal, hepatic, and cardiac impairments, those with epilepsy, pregnant or breast-feeding women, the elderly, and children.

Moreover, pharmacokinetic and/or pharmacodynamic tolerance effects resulting from chronic exposure to antidepressants have also been hypothesized to be related to the development of antidepressant tachyphylaxis or tolerance (loss of antidepressant efficacy when in maintenance treatment with the same drug and dosage regimen) [36, 37]. Noncompliance or nonadherence to drug treatment has also been associated with depressive symptomatology relapse [36, 37] and although this was not evaluated by patient plasma analysis, it was closely monitored by her husband and confirmed in each visit. Although these two factors could possibly be associated with the initial depressive relapse, in our patient this was probably more associated with the worsening of the underling depressive episode [36, 37].

## 4. Conclusions

This case report illustrates a relapse of depressive symptoms with insomnia that was successfully remitted after the addition of trazodone in a patient with treatment-resistant depression already performing a previous dual antidepressant combination of mirtazapine and paroxetine in standard antidepressant doses. The combination of trazodone as a third antidepressant in this case report not only allowed a rapid control of insomnia but also was efficacious for the management of other depressive symptoms and was well tolerated.

Trazodone addition as a triple antidepressant combination strategy may be used in treatment-resistant or recurrent depression patients in whom standard treatment options including dual antidepressant combination did not produce a successful response. Accordingly, combining trazodone with other antidepressants with complementary pharmacological actions can globally enhance antidepressant effect. This may be achieved by targeting residual symptoms not well treated by a single antidepressant effect such as SERT inhibition performed by SSRIs or through exerting other additional pharmacological actions. Interestingly, it may also resolve some tolerability issues by reducing SSRI or SNRI associated side-effects such as sexual dysfunction.

Moreover, combining trazodone with other antidepressants with complementary pharmacological actions might represent a more sustainable and tolerable option to the alternative of individually increasing the dose of the previous dual antidepressant combination or even other strategies including antidepressant augmentation (thyroid hormones, low doses of mood stabilizers, or antipsychotics) or ECT. In addition, it may also be a particularly useful treatment option in selected treatment-resistant depression cases presenting with insomnia in which the prescription of benzodiazepines and other sleep-inducers is not recommended.

However, some important pharmacologic issues should be considered when triple antidepressant combination is prescribed. Accordingly, antidepressant polypharmacy warrants a close clinical supervision and monitoring due to higher risk of pharmacokinetic and pharmacodynamic drug interactions of which it is essential to be aware. These may be associated with higher secondary side-effects and toxicity including the risk of serotonin syndrome. Prior to the addition and administration of a third antidepressant, an analytical check-up is therefore essential (including plasma glucose, FBC, U&E, and LFTs); thereafter, close, regular, and careful clinical monitoring including basic blood and biochemical reassessments during third antidepressant's titration should also be performed to prevent side-effects.

Other major limitations to this case report are intrinsically related to the naturalistic based observational study which does not allow the control and determination of the possible variables that may interfere with the treatment outcome. Further, patient adherence to treatment was not evaluated in plasma analysis and severity of depressive symptoms as well clinical response to treatment was not assessed with the aid of any clinical scale.

More investigation is needed to clearly define the place of triple antidepressant combination as a strategy in the management of treatment-resistant depression. Therefore, large, prospective, and well-controlled trials are needed for a rigorous establishment of risk-benefit ratio, as well as efficacy, tolerability, and safety issues and also for comparing directly with other existing alternatives.

## Figures and Tables

**Table 1:** Triple antidepressant combinations (drug family, active principle, and respective dosages) described in literature with good clinical outcomes (efficacy and tolerability).

Triple antidepressant combination	Study
SSRI (escitalopram 10 mg) + TCA (amitriptyline 25 mg) + NARI (reboxetine 2 mg)	Restifo [15]
TeCA (mianserin 20 mg) + TCA (nortriptyline 75 mg) + NARI (reboxetine 1 mg)	
SNRI (venlafaxine 150 mg) + NaSSA (mirtazapine 7.5 mg) + NARI (reboxetine 1 mg)	

MAOI (tranylcypromine 70 mg) + TCA (nortriptyline 100 mg) + SARI (trazodone 50 mg)	Thomas et al. [22]
MAOI (phenelzine 60 mg) + TCA (nortriptyline 150 mg) + SARI (trazodone 400 mg)	

SSRI (paroxetine 40 mg) + NaSSA (mirtazapine 30 mg) + SARI (trazodone 150 mg)	Present report

MAOI: monoamine oxidase inhibitor; NARI: noradrenaline reuptake inhibitor; NaSSA: noradrenergic and specific serotoninergic antidepressant; SARI: serotonin antagonist/reuptake inhibitor; SNRI: serotonin/noradrenaline reuptake inhibitor; SSRI: selective serotonin reuptake inhibitors; TCA: tricyclic antidepressants; TeCA: tetracyclic antidepressant.
